# Biogenesis of podosome rosettes through fission

**DOI:** 10.1038/s41598-017-18861-2

**Published:** 2018-01-11

**Authors:** Szu-Lin Kuo, Chien-Lin Chen, Yi-Ru Pan, Wen-Tai Chiu, Hong-Chen Chen

**Affiliations:** 1 Department of Life Sciences, National Chung Hsing University, Taichung, Taiwan; 2 Department of Biomedical Engineering, National Cheng Kung University, Tainan, Taiwan; 3 Institute of Biomedical Sciences, National Chung Hsing University, Taichung, Taiwan; 4 Institute of Biochemistry and Molecular Biology, National Yang-Ming University, Taipei, Taiwan

## Abstract

Podosomes are dynamic actin-based membrane protrusions that are important for extracellular matrix degradation and invasive cell motility. Individual podosomes are often found to organize into large rosette-like structures in some types of cells, such as osteoclasts, endothelial cells, Src-transformed fibroblasts, and certain highly invasive cancer cells. In this study, we show that new podosome rosettes arise through one of two mechanisms; *de novo* assembly or fission of a pre-existing podosome rosette in Src-transformed fibroblasts. Fission is a more efficient way than *de novo* assembly to generate new podosome rosettes in these cells. Podosome rosettes undergoing fission possess higher motility and a stronger matrix-degrading capability. Podosome rosette fission may be the result of polarized myosin II-mediated contractility of these structures, which is coordinately regulated by myosin light chain kinase and Rho-associated kinase II. Collectively, this study unveils a previously unknown mechanism—fission for the biogenesis of podosome rosettes.

## Introduction

Podosomes are highly dynamic, actin-rich adhesion structures that are found mainly in motile cells and are thought to contribute to tissue invasion and matrix remodeling^1^. Podosomes are dot-shaped structures with a diameter of 0.5–1 μm and a height of 0.2–5 μm, composed of a core of F-actin and actin regulators, such as cortactin and the Arp2/3 complex, surrounded by a ring structure containing integrins and scaffolding proteins, such as vinculin and talin^2^. The podosomes recruit matrix metalloproteases and facilitate focal degradation of extracellular matrix (ECM) and invasion^3^. Many invasive cancer cells display structures similar to podosomes, called invadopodia, that represent the major sites of ECM degradation in these cells^4^. Many regulators of podosome turnover have been identified, including tyrosine kinases, Rho GTPases, actin regulators, and the microtubule system^5^.

Podosomes can serve as the structural unit for superstructures, such as podosome clusters, rosettes, or belts. Podosomes rosettes with a diameter of 5–20 μm are often found in Src-transformed fibroblasts^6,7^, osteoclasts^8^, endothelial cells^9,10^, and some highly invasive cancer cells^7,11^. Podosome rosettes are much more potent than podosome dots for promoting matrix degradation^7^. However, the mechanism for the self-organization of podosomes remains elusive. Additional regulators are believed to be required for the assembly of higher-ordered podosome structures. For example, the cytoplasmic tyrosine kinase FAK is dispensable for podosome dots, but it is required for the assembly of podosome rosettes^7^.

Moreover, within higher-ordered podosome clusters, individual podosome cores seem connected by unbranched actin filaments^8^. Non-muscle myosin II activity was shown to be important for the integrity of individual podosomes in dendritic cells^12^ and the formation of ring-like clusters of podosomes in Rous sarcoma virus-transformed baby hamster kidney cells^13^. Recently, the formin FHOD1 was shown to regulate the actomyosin-based contractility of podosome-connecting actin filaments, thus regulating the connectivity of podosomes in podosome clusters in primary macrophages^14^. In this study, we surprisingly found that new podosome rosettes can be generated by fission of pre-existing podosome rosettes in Src-transformed fibroblasts. This phenomenon has never been described and represents a novel mechanism for the biogenesis of podosome rosettes.

## Results

### Podosomes rosettes can be generated through *de novo* assembly and fission

**Figure 1:**
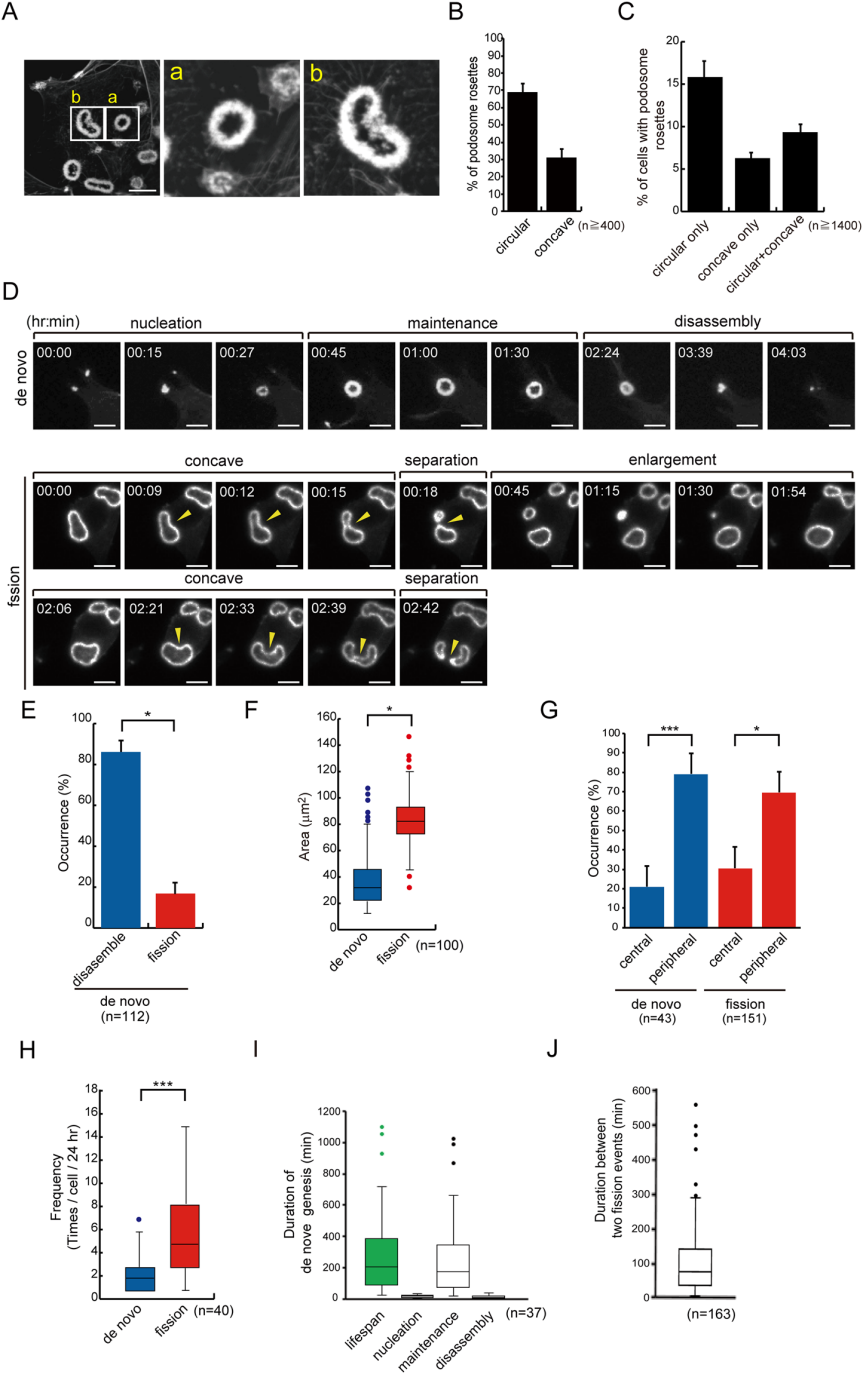
Podosome rosettes can be generated through *de novo* and fission in Src-transformed NIH3T3 cells. (**A**) SrcY527F-transformed NIH3T3 cells were fixed and stained for actin filaments with phalloidin. Images of podosome rosettes with circular (a) or concave (b) shape were taken with Zeiss confocal microscopy. The scale bar represents 10 μm. (**B**) The percentage of circular- or concave-type podosome rosettes in total counted podosome rosettes was measured. Values (means ± s.d.) are from three independent experiments. (**C**) The percentage of cells with podosome rosettes was measured. Values (means ± s.d.) are from three independent experiments. (**D**) GFP-UtrCH was transiently expressed in SrcY527F-transformed 3T3 cells and the cells were monitored with time-lapse fluorescence microscopy. Representative image frames are shown to demonstrate that new podosome rosettes arise through one of two mechanisms; *de novo* assembly or fission. The arrowheads indicate the concave of podosome rosettes undergoing fission. The scale bar represents 10 μm. (**E**) The percentage of *de novo* assembled podosome rosettes that were eventually disassembled or underwent fission was measured with time-lapse fluorescence microscopy. Values (means ± s.d.) are from three independent experiments. (**F**) The average size of the podosome rosette as they started to undergo fission or disassembly was measured with ZEISS ZEN2 software. The results are expressed as box-and-whisker plots. *P < 0.05. (**G**) The percentage of *de novo* assembly or fission of podosome rosettes occurred within the 5-μm range from the cell periphery or the rest area of the cell (cell center) was measured with time-lapse fluorescence microscopy. Values (means ± s.d.) are from three independent experiments. *P < 0.05; ***P < 0.005. (**H**) The frequencies of cells to generate podosome rosettes through *de novo* assembly or fission were measured with time-lapse fluorescence microscopy. The results are expressed as box-and-whisker plots. ***P < 0.005. (**I**) The lifespan of podosome rosettes generated through *de novo* assembly was measured with time-lapse fluorescence microscopy, which can be divided into three phases, *i.e*. nucleation, maintenance, and disassembly. The results are expressed as box-and-whisker plots. (**J**) The duration between two consecutive fission events was measured with time-lapse fluorescence microscopy. The results are expressed as box-and-whisker plots.

Src-transformed fibroblasts have been used as a model to study the assembly of podosome rosettes^7,15^. In this study, we noticed that not all of podosome rosettes displayed a perfect circular structure in Src-transformed NIH3T3 fibroblasts (Fig. 1A). Instead, approximately 30% of podosome rosettes showed a single or double concave appearance (Fig. 1B). Podosome rosettes were detected in approximately 30% of the cells, half of which contained concave ones (Fig. 1C). Both circular and concave types of podosome rosettes can be detected with total internal reflection fluorescence microscopy (Fig. S1), indicating that they are in close proximity to the ventral surface of the cell.

To examine the fate of concave podosome rosettes, Src-transformed NIH3T3 fibroblasts were transiently transfected with green fluorescent protein (GFP)-UtrCH (calponin homology domain of utrophin) or GFP-actin and monitored with time-lapse fluorescence microscopy. Surprisingly, we found that substantial amounts of concave podosome rosettes underwent “fission” to generate daughter podosome rosettes (Fig. 1D). This phenomenon was also observed in Src-transformed mouse embryonic fibroblasts (Fig. S2A) and lung cancer CL1–5 cells (Fig. S2B). The fission was manifested by enlargement, concave and eventual cleavage of a pre-existing podosome rosette into daughter podosome rosettes (Fig. 1D). Therefore, concave appears to be a hallmark of podosome rosette fission. The majority of *de novo* assembled podosome rosettes were eventually disassembled, but approximately 16% of which underwent fission (Fig. 1E). The average size of the podosome rosette as they started to undergo fission was approximately 80 μm^2^, which was 2-fold larger than the size of the podosome rosette as they started to disassemble (Fig. 1F). The “*de novo* assembly” and “fission” of podosome rosettes were largely occurred within the 5-μm range from the cell periphery (Fig. 1G). However, the frequency of “*de novo* assembly” and “fission” of podosome rosettes were 2.38 ± 0.84 and 6.45 ± 0.83 per cell over 24 h, respectively (Fig. 1H), indicating that fission occurred more frequently than *de novo* assembly for the biogenesis of podosome rosettes in Src-transformed NIH3T3 fibroblasts. Moreover, the lifespan of podosome rosettes through *de novo* assembly ranged between 87 min and 387 min (first and third quartiles of 37 data) with a median 204 min (Fig. 1I), which can be divided into three phases, *i.e*. nucleation, maintenance, and disassembly (Fig. 1D and I). The duration between two consecutive fission events ranged between 42 min and 144 min (first and third quartiles of 163 data) with a median 78 min (Fig. 1J), which reflects the period of time required for the biogenesis of podosome rosettes through fission. The duration for *de novo* assembly was approximately 2.6-fold longer than for fission (Fig. 1I and J), indicating that fission is a more efficient way than assembly to generate new podosome rosettes.

### Podosome rosettes undergoing fission possess higher motility and matrix-degrading capability than non-fission podosome rosettes

**Figure 2:**
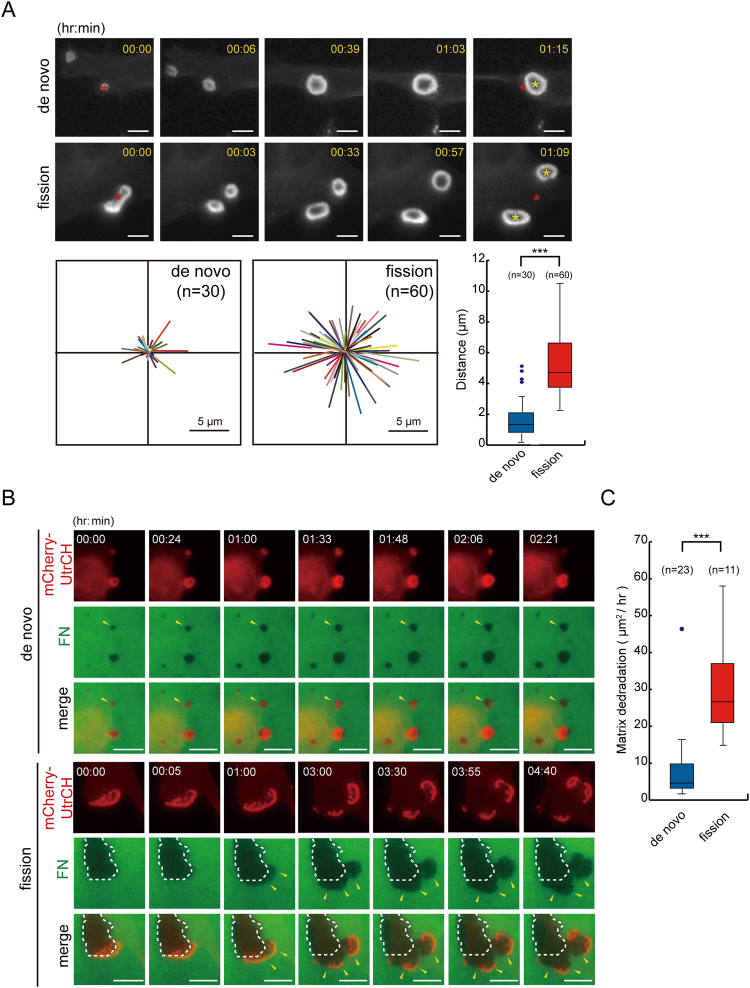
Podosome rosettes undergoing fission possess higher motility and a stronger matrix-degrading capability than non-fission podosome rosettes. (**A**) SrcY527F-transformed NIH3T3 cells transiently expressing GFP-UtrCH were monitored with time-lapse fluorescence microscopy. Representative image frames are shown to illustrate the movement of podosome rosettes. The sites of podosome rosettes before and after tracking are marked by red and yellow asterisks, respectively. The scale bar represents 10 μm. The movement tracks of non-fission and fission podosome rosettes are plotted. The movement distance of podosome rosette per 30 min was measured. The results are expressed as box-and-whisker plots. ***P < 0.005. (**B**) SrcY527F-transformed NIH3T3 cells transiently expressing mCherry-UtrCH were plated on Alexa Fluor 488-labeled fibronectin (FN) for 12 h. The degraded areas by podosome rosettes were monitored with time-lapse fluorescence microscopy for 24 h. The arrowheads indicate the areas which were degraded during our recording. The dashed lines enclose the areas which were degraded prior to our recording. The scale bar represents 10 μm. (**C**) The average degraded areas by podosome rosettes undergoing de novo assembly or fission were measured. The results are expressed as box-and-whisker plots. ***P < 0.005.

The movement of podosome rosettes was recorded with time-lapse fluorescence microscopy. We found that non-fission podosome rosettes tended to localize within a region proximal to the site where it was originally assembled (Fig. 2A). In contrast, podosome rosettes undergoing fission had much higher motility, which allowed them to depart from their original location (Fig. 2A). More importantly, podosome rosettes undergoing fission were able to degrade ECM proteins along their movement, whereas circular podosome rosettes degraded ECM proteins *in situ* (Fig. 2B). Consequently, the areas of ECM proteins degraded by podosome rosettes undergoing fission were larger than those degraded by non-fission podosome rosettes (Fig. 2C). These results indicate that fission facilitates the movement and matrix degradation of podosome rosettes.

### Podosome rosette fission may be the result of polarized myosin II-mediated contractility at these structures

**Figure 3:**
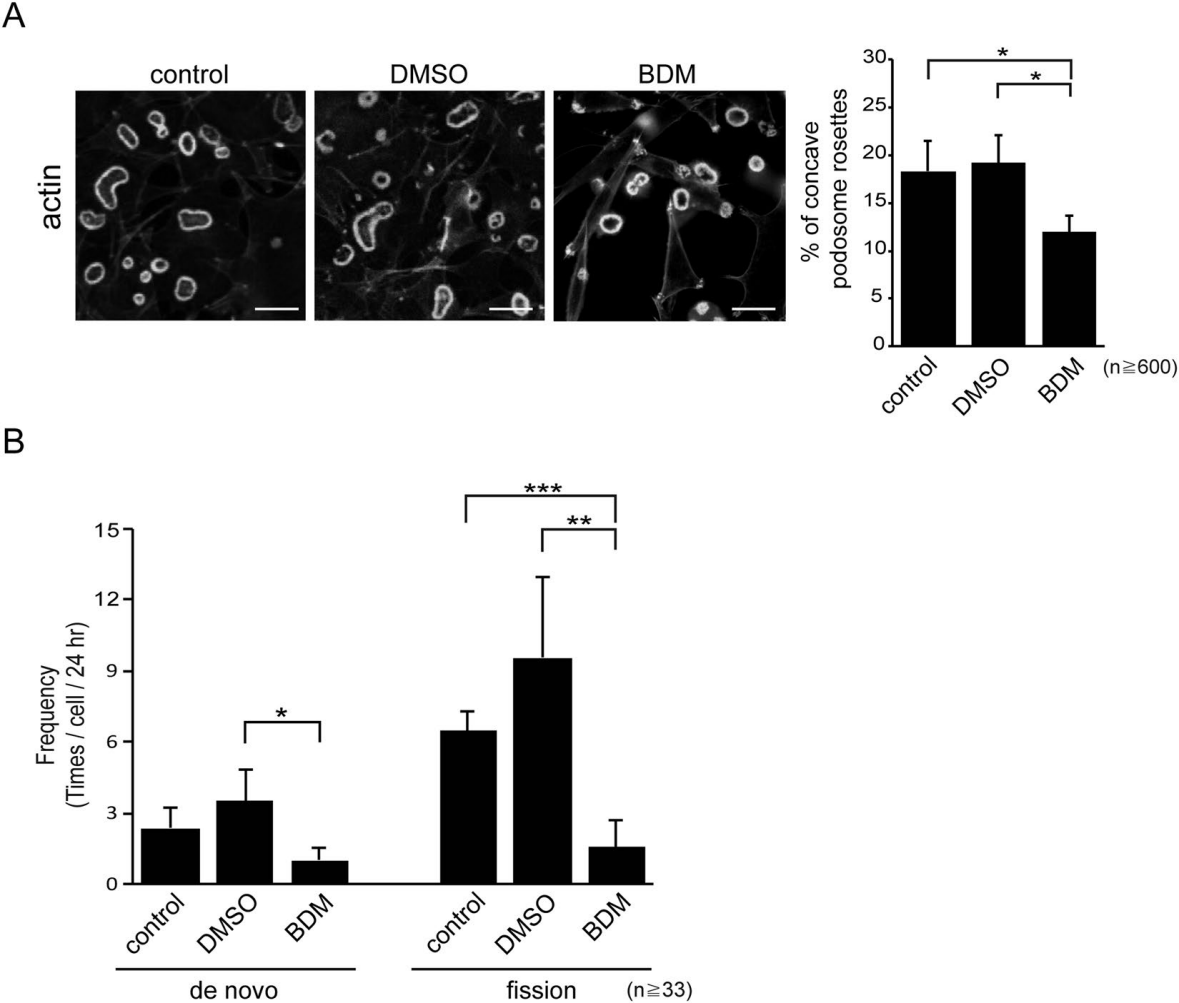
Myosin II activity is required for biogenesis of podosome rosettes. (**A**) SrcY527F-transformed NIH3T3 cells were treated with 15 μM of the myosin II inhibitor BDM or dimethyl sulfoxide (DMSO) as the solvent control for 24 h. The cells were fixed and stained for actin filaments with phalloidin. Images were taken with Zeiss Apotome microscopy. The scale bar represents 10 μm. The percentage of concave podosome rosettes in total counted podosome rosettes was measured. Values (means ± s.d.) are from three independent experiments. *P < 0.05. (**B**) GFP-UtrCH was transiently expressed in SrcY527F-transformed NIH3T3 cells and the cells were monitored with time-lapse fluorescence microscopy in the presence of 15 μM BDM or DMSO for 24 h. The frequencies of the cell to generate podosome rosettes through de novo assembly and fission were measured. Values (means ± s.d.) are from three independent experiments. *P < 0.05; **P < 0.001; ***P < 0.005.

**Figure 4:**
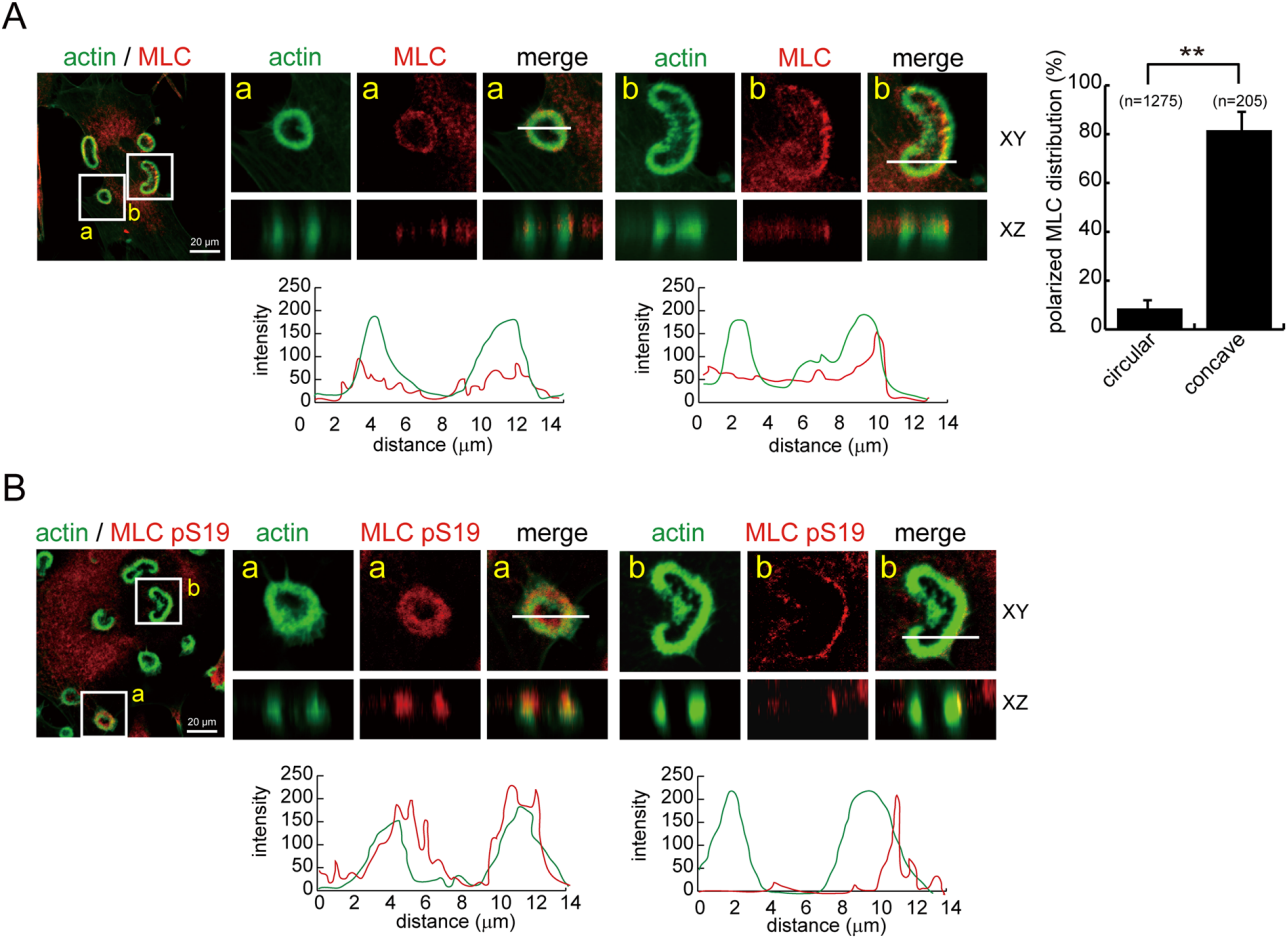
Polarized distribution of MLC and phospho-MLC in concave podosome rosettes. (**A**) SrcY527F-transformed NIH3T3 cells were fixed and stained for actin filaments with phalloidin and MLC with anti-MLC. The z-stack images were obtained and reconstituted with confocal microscopy. Representative images of podosome rosettes with circular (a) or concave (b) shape are shown. Polarized distribution of MLC in circular-type or concave-type podosome rosettes was measured. Values (means ± s.d.) are from three independent experiments. **P < 0.01. Graphs show the relative fluorescence intensity of the lines that were scanned by confocal microscopy. (**B**) The cells were fixed and stained for actin filaments with phalloidin and S19-phosphorylated MLC with the anti-MLC pS19. Representative images of podosome rosettes with circular (a) or concave (b) shape are shown. Graphs show the relative fluorescence intensity of the lines that were scanned by confocal microscopy.

**Figure 5:**
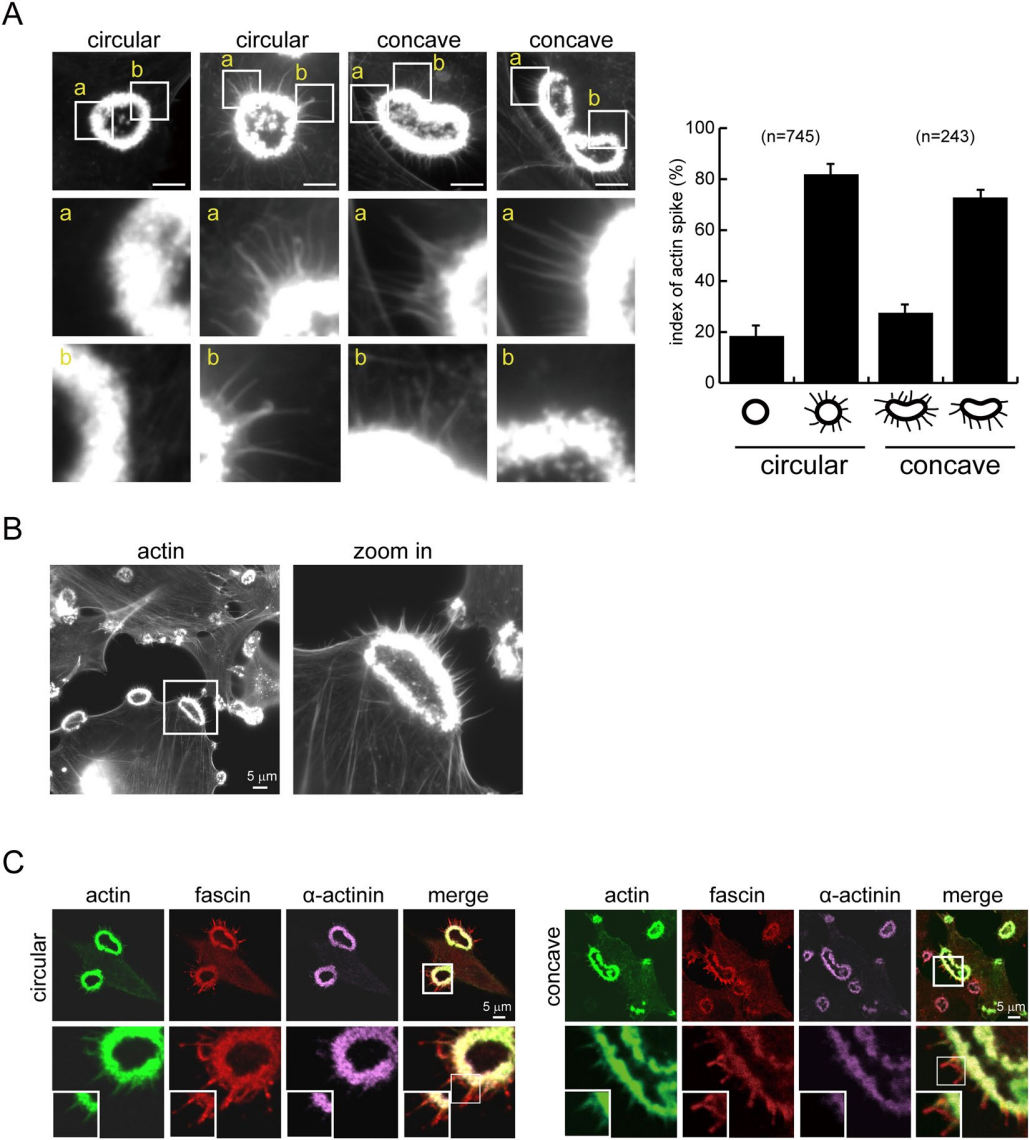
The actin spikes at the convex of podosome rosettes are positioned towards to the plasma membrane. (**A**) The cells were stained for actin filaments with phalloidin and the images were taken with Zeiss ApoTome microscopy. Representative images of podosome rosettes with circular or concave morphology are shown. The scale bar represents 10 μm. The distribution of actin spikes at circular-type or concave-type podosome rosettes was measured. Values (means ± s.d.) are from three independent experiments. (**B**) The representative image shows the actin spikes protrude towards to the plasma membrane. The scale bar represents 5 μm. (**C**) SrcY527F-transformed 3T3 cells transiently expressing GFP-UtrCH were fixed and stained for fascin (red) and α-actinin (purple). Images were taken with confocal microscopy. Note that fascin, but not α-actinin, is localized at the actin spike.

We found that 2,3-butanedione monoxime (BDM), an inhibitor of myosin ATPase, decreased the ratio of concave podosome rosettes (Fig. 3A). Importantly, the frequencies of both “*de novo* assembly” and “fission” of podosome rosettes were suppressed by BDM (Fig. 3B), indicating that actomyosin-mediated contractility is involved in both processes. We found that myosin light chain (MLC) and its phosphorylated form were distributed mainly at the convex of concave-type podosome rosettes (Fig. 4). In addition, the actin spikes protruding from the main F-actin structure of podosome rosettes were detected mainly at the convex of concave-type podosome rosettes (Fig. 5A), ~82% of which were positioned towards to the plasma membrane (Fig. 5B). Interestingly, both fascin (a parallel F-actin bundling protein) and α-actinin (an anti-parallel F-actin bundling protein) were localized at podosome rosettes, yet only fascin was detected in the actin spikes (Fig. 5C), indicating that podosome rosettes contain both parallel and anti-parallel F-actin, whereas the surrounding actin spikes are composed only of parallel F-actin. These results together suggest that polarized distribution of MLC and actin spikes may cause polarized contractility, which leads to concave and eventually fission of podosome rosettes.

### Podosome rosette fission is coordinately regulated by MLCK and ROCK

**Figure 6:**
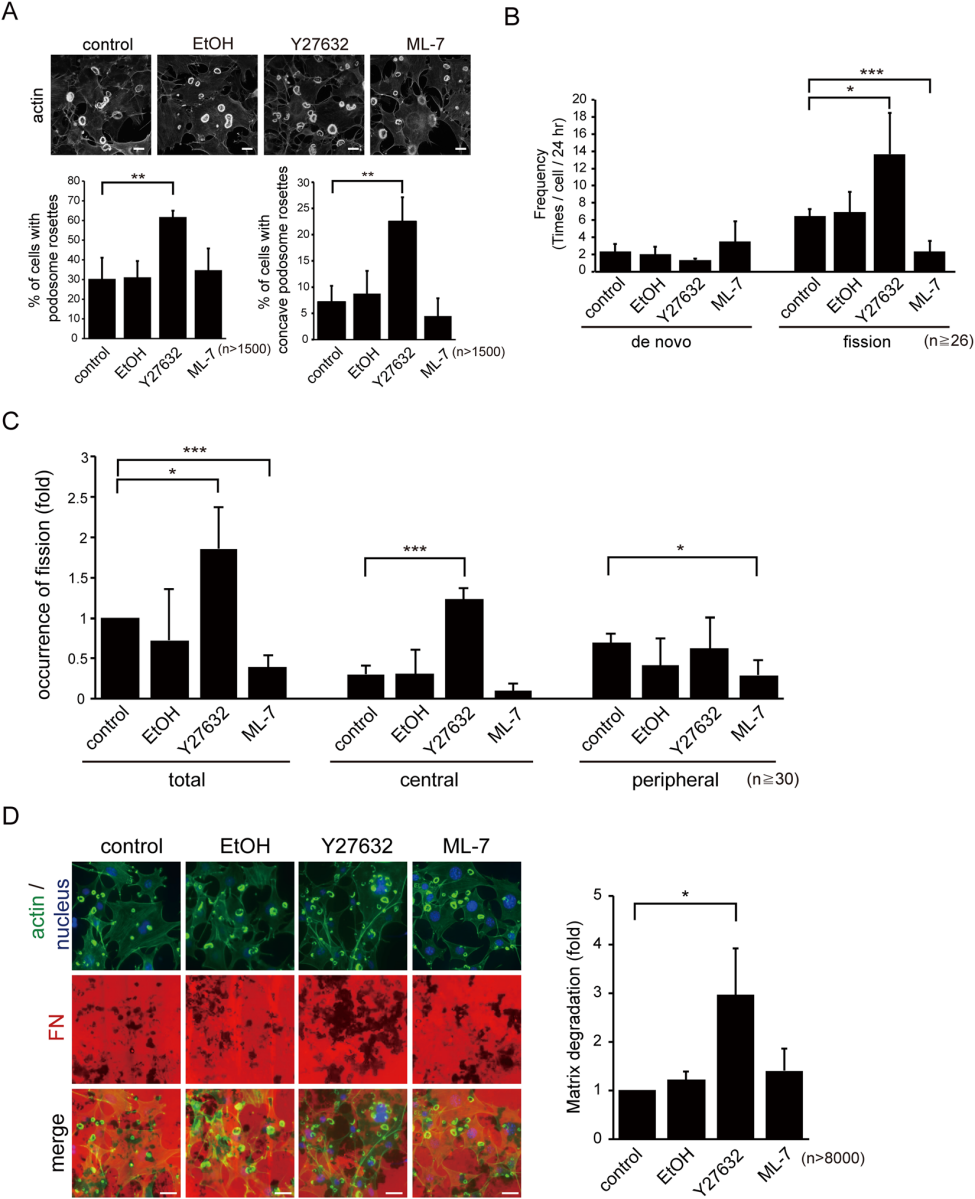
Podosome rosette fission was inhibited by ML-7, but increased by Y27632. (**A**) SrcY527F-transformed 3T3 cells were treated with 10 μM of the ROCK inhibitor Y27632 or the MLCK inhibitor ML-7 for 3 h. Ethanol (EtOH) was used as the solvent control. The cells were fixed and stained for actin filaments with phalloidin. Representative images were taken with epifluorescence microscopy. The percentage of the cells containing podosome rosettes (left) and the percentage of the cells containing concave podosome rosettes (right) were measured. The scale bar represents 20 μm. Values (means ± s.d.) are from three independent experiments. **P < 0.01; ***P < 0.005. (**B**) GFP-UtrCH was transiently expressed in SrcY527F-transformed 3T3 cells and monitored with time-lapse microscopy for 24 h. The frequencies of cells to generate podosome rosettes through *de novo* assembly and fission were measured. Values (means ± s.d.) are from three independent experiments. *P < 0.05. ***P < 0.005. (**C**) The cells were transiently transfected with GFP-UtrCH and monitored in the presence of 10 μM of Y27632 of ML-7 with time-lapse microscopy for 24 h. The occurrence of podosome rosette fission within the 5-μm range from the cell periphery or the rest area of the cell (cell center) was measured. Values (means ± s.d.) are from three independent experiments and expressed as -fold relative to the control. *P < 0.05; ***P < 0.005. (**D**) SrcY527F-transformed 3T3 cells were plated on Alexa Fluor 546-conjugated fibronectin (FN) in the presence of 10 μM of Y27632 of ML-7 for 24 h, and the cells were stained with phalloidin. Images were taken with epifluorescence microscopy. The average degraded areas by the cells were measured and expressed as fold relative to the control. The scale bar represents 20 μm. Values (means ± s.d.) are from three independent experiments. *P < 0.05.

**Figure 7:**
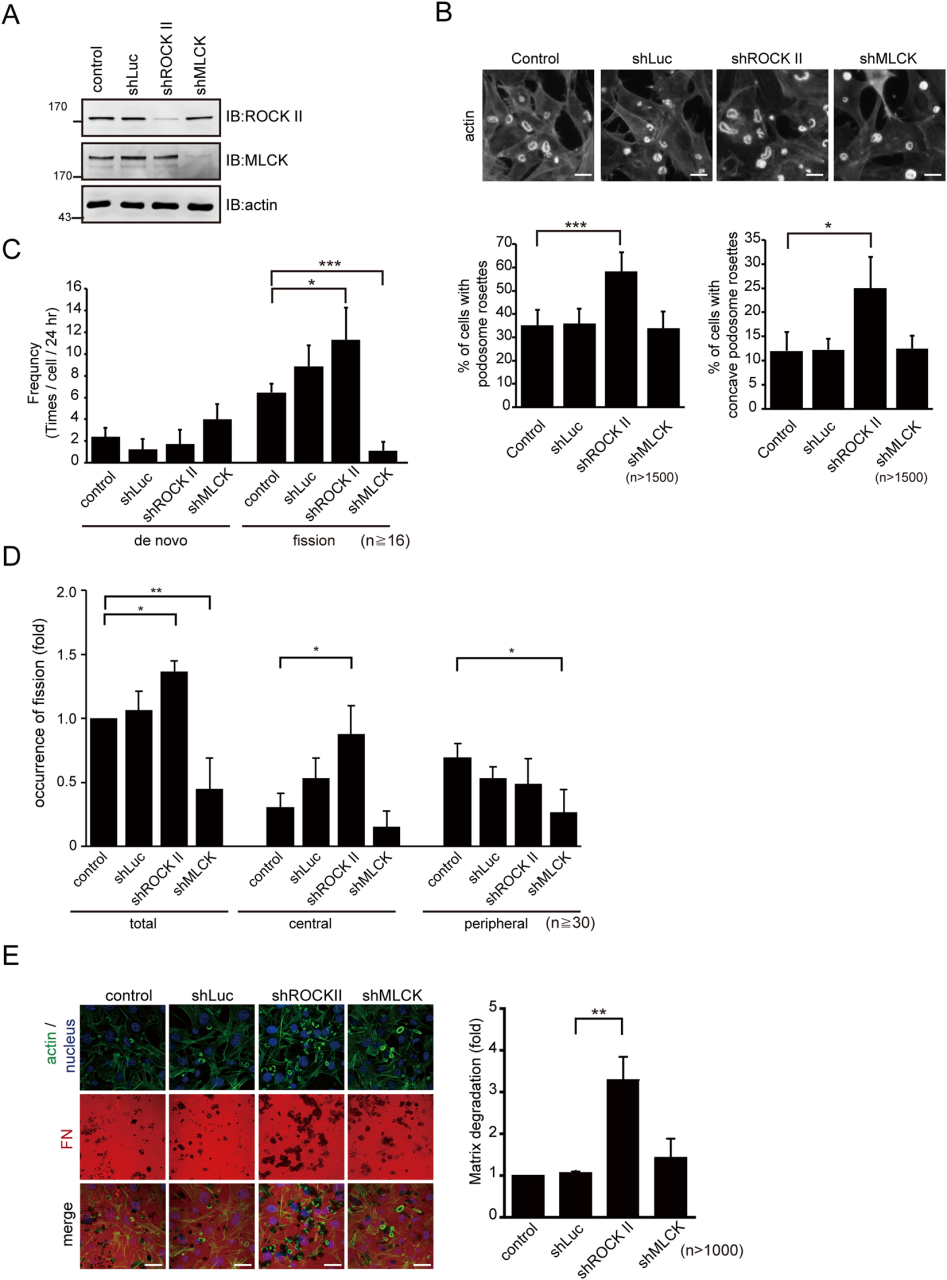
Podosome rosette fission was suppressed by MLCK knockdown, but enhanced by ROCK II knockdown. (**A**) SrcY527F-transformed 3T3 cells were infected with lentiviruses expressing shRNAs specific to MLCK, ROCK II, or luciferase as a control and selected in the medium containing puromycin. The cell lysates were analyzed by immunoblotting with antibodies specific to ROCK II and MLCK. (**B**) The cells expressing shRNAs were fixed and stained for actin filaments with phalloidin. Representative images were taken with epifluorescence microscopy. The scale bar represents 20 μm. The percentage of the cells containing podosome rosettes (left) and the percentage of the cells containing concave podosome rosettes (right) were measured. Values (means ± s.d.) are from three independent experiments. *P < 0.05; ***P < 0.005. (**C**) The cells expressing shRNAs were transiently transfected with GFP-UtrCH and monitored with time-lapse microscopy for 24 h. The frequency of the cell to generate podosome rosettes by *de novo* assembly and fission was measured. Values (means ± s.d.) are from three independent experiments. *P < 0.05; ***P < 0.005. (**D**) The cells expressing shRNAs were transiently transfected with GFP-UtrCH and monitored with time-lapse microscopy for 24 h. The occurrence of podosome rosette fission within the 5-μm range from the cell periphery or the rest area of the cell (cell center) was measured. Values (means ± s.d.) are from three independent experiments and expressed as -fold relative to the control. *P < 0.05; **P < 0.01. (**E**) The cells expressing shRNAs were plated on Alexa Fluor 546-conjugated fibronectin for 24 h and stained for actin filaments and nuclei. Images were taken with confocal microscopy. The degraded areas by the cells were measured and expressed as fold relative to the control. The scale bar represents 20 μm. Values (means ± s.d.) are from three independent experiments. **P < 0.01.

MLC kinase (MLCK) and Rho-associated kinase (ROCK) have been shown to regulate cellular contractility^16,17^. Both kinases were found to localize at podosome rosettes (Fig. S3). Remarkably, the percentage of cells with concave podosome rosettes (Fig. 6A) and the frequency of podosome rosette fission were apparently increased by the ROCK inhibitor Y27632, but decreased by the MLCK inhibitor ML-7 (Fig. 6B). Y27632 promoted the fission of podosome rosettes at the cell center, whereas ML-7 inhibited the fission at the cell periphery (Fig. 6C). Accordingly, the depletion of ROCK II, but not ROCK I (Fig. S4), by short-hairpin RNAs (shRNAs) increased the percentage of cells with concave podosome rosettes (Fig. 7A and B) as well as the frequency of podosome rosette fission (Fig. 7C). In contrast, depletion of MLCK decreased the frequency of podosome rosette fission (Fig. 7C). Spatially, depletion of MLCK suppressed the fission at the cell periphery, whereas depletion of ROCK II promoted the fission at the cell center (Fig. 7D). The increase in podosome rosette fission by the suppression of ROCK II was correlated with increased matrix degradation of the cells (Fig. 6D and E). These results together suggest a positive role for MLCK and a negative role for ROCK in podosome rosette fission.

### Enforced expression of MLCK or ROCK II inhibits the formation of podosome rosettes

**Figure 8:**
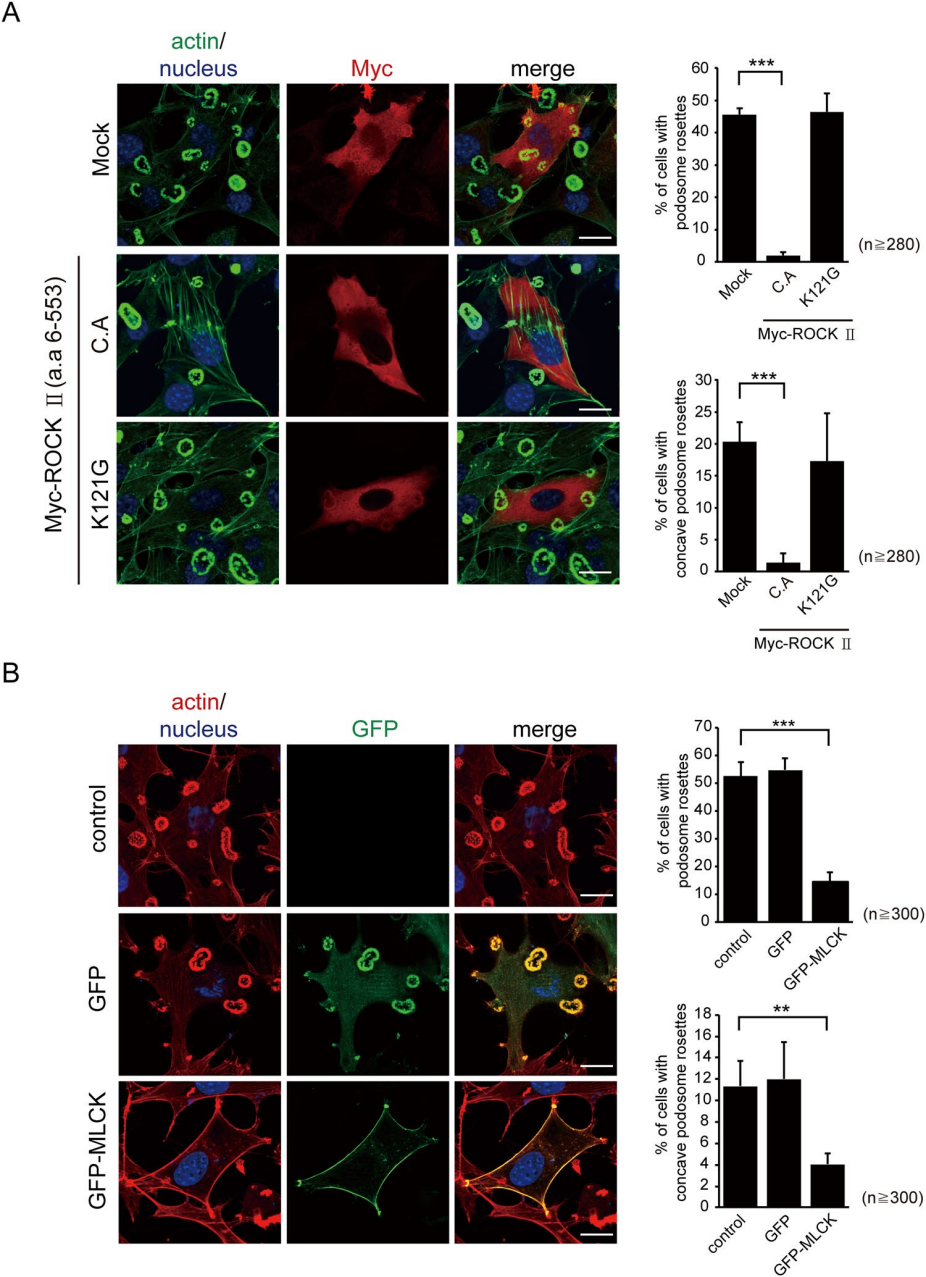
Enforced expression of ROCK II or MLCK inhibited the formation of podosome rosettes. (**A**) Myc-tagged ROCK II (constitutively active or catalytically deficient K121G mutant) was transiently expressed in SrcY527F-transformed NIH3T3 cells. The cells were fixed and stained for actin filaments with phalloidin and for Myc-tagged ROCK II with anti-Myc. Images were taken with Zeiss Apotome microscopy. The scale bar represents 20 µm. The percentage of the cells containing podosome rosettes and the percentage of the cells containing concave podosome rosettes were measured. Values (means ± s.d.) are from three independent experiments. ***P < 0.005. (**B**) GFP-MLCK or GFP alone was transiently expressed in SrcY527F-transformed NIH3T3 cells. The cells were fixed and stained for actin filaments and nuclei. Images were taken with Zeiss Apotome microscopy. The scale bar represents 20 µm. The percentage of the cells containing podosome rosettes and the percentage of the cells containing concave podosome rosettes were measured. Values (means ± s.d.) are from three independent experiments. **P < 0.01; ***P < 0.005.

The effect of overexpression of MLCK or ROCK II on the biogenesis of podosome rosettes was examined in Src-transformed NIH3T3 fibroblasts. We found that transient expression of constitutively active ROCK II, but not the catalytically deficient K121G mutant, inhibited the formation of podosome rosettes, which was accompanied by enhanced formation of stress fibers (Fig. 8A). In addition, transient expression of GFP-MLCK inhibited the formation of podosome rosettes, but enhanced the formation of the membrane cortical F-actin (Fig. 8B). These results suggest that enforced formation of stress fibers and cortical actin cytoskeleton by ROCK and MLCK, respectively, counteracts the formation of podosome rosettes.

## Discussion

The question of how podosome rosettes are formed has been studied for more than 30 years. It is generally believed that podosome rosettes are organized by self-assembly of individual podosomes. In this study, we reported for the first time that daughter podosome rosettes can be generated by fission of pre-existing mother podosome rosettes. In Src-transformed NIH3T3 fibroblasts, new podosome rosettes arise more frequently by fission than by *de novo* assembly. Podosome rosettes undergoing fission are characterized by concave shape, higher motility, and stronger matrix-degrading capability (Figs 1 and 2). Our results demonstrate that fission is a more efficient way than *de novo* assembly to generate new podosome rosettes and to facilitate cell invasion.

The myosin II inhibitor BDM inhibited both “de novo assembly” and “fission” of podosome rosettes (Fig. 3B), indicating that actomyosin-mediated contractility is involved in both processes. In addition, the treatment of BDM led to small, non-concave and radially symmetric rosettes (Fig. 3A). This indicates that the myosin II activity is required for the growth of podosome rosettes and supports that the growth of podosome rosettes to a certain size is necessary prior to concave and fission. The necessity of myosin II activity (Fig. 3) and the polarized distributions of MLC (Fig. 4) and actin spikes (Fig. 5) at the convex of concave-type podosome rosettes suggest that podosome rosette fission may be the result of polarized myosin II-mediated contractility of these structures.

Both fascin (a parallel F-actin bundling protein) and α-actinin (an anti-parallel F-actin bundling protein) were detected in the main structure of podosome rosettes (Fig. 5C), which may promote the fusion of individual podosomes and facilitate the non-polarized contractility at the main structure of the rosette. In contrast, only fascin was detected in the actin spikes (Fig. 5C), indicating that the actin spikes are parallel F-actin structure. Moreover, the actin spikes protruding from the main F-actin structure of podosome rosettes were mainly detected at the convex of concave-type podosome rosettes (Fig. 5A), ~82% of which were positioned towards to the plasma membrane similar to the structure of filopodia (Fig. 5B). It is possible that the actin spikes may initially protrude from the main F-actin structure of podosome rosette without polarity until they link to the plasma membrane through some linker proteins. The linkage of those actin spikes to the plasma membrane and subsequently make contact to extracellular matrix may generate a pulling force as the cell membrane extends outwards for cell motility, which thereby triggers a morphological change of podosome rosettes and a polarized distribution of myosin. Larger podosome rosettes may facilitate the bending through the pulling force generated by the actin spikes.

In this study, we demonstrated that podosome rosette fission is regulated positively by MLCK, but negatively by ROCK II (Figs 6 and 7). Although ROCK I and ROCK II have been shown to regulates invadopodia activity through different signaling pathways in certain types of cancer cells^18^, we show here that ROCK I is not involved in the podosome rosette fission in Src-transformed NIH3T3 cells (Fig. S4). Given MLCK and ROCK II can regulate MLC phosphorylation^19,20^, why do they play distinct roles in podosome rosette fission? MLCK and ROCK II have been shown to play distinct roles in the spatial regulation of MLC phosphorylation for assembly of focal adhesions and stress fibers in 3T3 fibroblasts^20^. At the cell periphery, MLCK but not ROCK II appears to be the kinase responsible for phosphorylating MLC^20^. We found that the inhibition of MLCK suppressed the fission of podosome rosettes at the cell periphery, whereas the inhibition of ROCK II promoted the fission at the cell center in Src-transformed 3T3 fibroblasts (Figs 6 and 7). Therefore, the distinct roles for MLCK and ROCK II in podosome rosette fission could be due to their distinct spatial regulation of MLC phosphorylation. Moreover, we found that enforced expression of ROCK II and MLCK respectively led to the assembly of stress fibers in the center of cells and the membrane cortical F-actin at the cell periphery, both of which abrogated podosome rosette formation in Src-transformed 3T3 fibroblasts (Fig. 8). These results suggest that abnormal cellular contractility induced by aberrant activation of ROCK II and/or MLCK may prevent the formation of podosome rosettes.

Alternatively, ROCK II may suppress podosome rosette fission through other proteins rather than MLC. Compared to MLCK, ROCK II has a broader range of downstream substrates^21^. For example, ROCK II can activate LIM kinase which then phosphorylates and inhibits cofilin^21,22^. Cofilin is an actin severing protein that is known to regulate cytoskeleton architecture, cell migration and invasion^23^. Because cofilin has been known to be required during podosome pattering in osteoclasts^24^, it is possible that ROCK II may suppress podosome rosette fission through its inhibition of cofilin.

In addition to Src-transformed fibroblasts, podosome rosette fission was also observed in lung cancer CL1–5 cells (Fig. S2). It is not known whether other types of cells, such as endothelial cells and osteoclasts, form podosome rosettes through fission. If podosome rosette fission does occur in those cells, how do those cells respond to extracellular cues to regulate this process? ECM rigidity and growth factors have been reported to affect the assembly of podosome rosettes^10,25–27^. It will be of interest to examine whether podosome rosette fission is regulated by those factors.

## Materials and Methods

### Materials

Polyclonal anti-ROCK I (H-85) and anti-ROCK II (H-85) antibodies and monoclonal anti-Myc (9E10) antibody were purchased from Santa Cruz Biotechnology (Santa Cruz, CA). Polyclonal anti-α-actinin antibody and monoclonal anti-MLC pS19 antibody were purchased from Cell Signaling Technology (Danvers, MA). Monoclonal anti-MLC (MY-21) antibody, monoclonal anti-MLCK (K-36) antibody, gelatin, BDM, and ML-7 were purchased from Sigma-Aldrich (St. Louis, MO). Monoclonal anti-facsin antibody, Y27632, puromycin, and fibronectin were purchased from EMD Millipore (Billerica, MA). Alexa Fluor 488-phalloidin, the Alexa Fluor 546 protein labeling kit, Alexa Fluor 488- and Alexa Fluor 546-conjugated secondary antibodies, and Lipofectamine were purchased from Thermo Fisher Scientific (Waltham, MA). The horseradish peroxidase-conjugated goat anti-mouse and anti-rabbit antibodies were purchased from Jackson ImmunoResearch Laboratories (West Grove, PA). The plasmid pCS2+ -GFP-UtrCH was purchased from Addgene (Cambridge, MA). The plasmid pEGFP-actin was purchased from Takara Bio USA, Inc. (Mountain View, CA). The plasmid pEGFP-N1-MLCK was described previously^28^. The plasmids mCherry-UtrCH, pEF-Myc-ROCK II C.A. (a.a 6–553), and pEF-Myc-ROCK II K121G (a.a 6–553) were kindly provided by Dr. H. H. Lee (National Yang-Ming University, Taiwan).

### Cell Culture and Transfections

SrcY527F-transformed NIH3T3 cells, v-Src-transformed MEFs, and human lung cancer CL1–5 cells were maintained as previously described^7^. For transient transfection, cells were seeded on 60-mm culture dishes for 18 h and were then transfected with plasmids using Lipofectamine

### shRNA-mediated Knockdown

The lentiviral expression system and the pLKO-AS1-puro plasmid encoding shRNAs were obtained from the National RNAi Core Facility, Academia Sinica, Taiwan. The target sequences for ROCK II and MLCK were 5-GCATCTCTTGAAGAAACAAAT-3 (mouse) and 5- GCGGGAGTGTATCAAGTACAT-3 (mouse), respectively. The target sequences for ROCK I #1 and #2 were 5-CGGGAGTTACAAGATCAACTT-3 (mouse) and 5-GCAGTGTCTCAAATTGAGAAA (mouse). To produce lentiviruses, HEK293T cells were co-transfected with pCMV-ΔR8.91 (2.25 μg), pMD.G (0.25 μg), and pLKO-AS1-puro-shRNA using LipofectAMINE. After 3 days, the medium containing lentivirus particles was collected and stored at −80 °C. The cells were infected with lentiviruses encoding shRNAs in the presence of 8 μg/ml polybrene for 24 h and were subsequently selected in the growth medium containing 2 μg/ml puromycin for 3 days.

### Immunoblotting

To prepare whole cell lysates, cells were lysed in 1% Nonidet P-40 lysis buffer (1% Nonidet P-40, 20 mM Tris-HCl, pH 8.0, 137 mM NaCl, 10% glycerol, and 1 mM Na_3_VO_4_) containing protease inhibitors (phenylmethylsulfonyl fluoride, aprotinin, and leupeptin). Whole cell lysates were boiled for 3 min in SDS sample buffer, subjected to SDS-polyacrylamide gel electrophoresis, and transferred to nitrocellulose (Schleicher and Schuell). Immunoblotting was carried out with the indicated antibodies using the Millipore Immobilon Western chemiluminescent HRP substrate for detection. Chemiluminescent signals were detected with a Fuji LAS-4000 luminescence imaging system.

### Matrix Degradation Assay

Alexa Fluor 488 or 546–conjugated fibronectin was prepared according to the manufacturer’s instructions (Thermo Fisher Scientific). Cells were plated on glass coverslips coated with 20 ng/ml Alexa Fluor–conjugated fibronectin. After 24 h or 36 h, the cells were fixed and stained for F-actin and nuclei. The areas in which Alexa Fluor–conjugated fibronectin were degraded by podosome rosettes were measured using Adobe Photoshop.

### Fluorescence Microscopy

Alexa Fluor 488-conjugated phalloidin was used to stain actin filaments. For immunofluorescent staining, the cells were fixed with 4% paraformaldehyde in phosphate-buffered saline for 30 min at room temperature and permeabilized with 0.5% Triton X-100 in phosphate-buffered saline for 15 min at room temperature. In Fig. 4D, the cells were fixed with 3% paraformaldehyde in 90% methanol. The primary antibodies used in immunofluorescent staining in this study were anti-Myc (1:200), anti-MLC (1:100), anti-MLC pS19 (1:100), anti-MLCK (1:200), anti-ROCK II (1:200). anti-facsin (1:100), and anti-α-actinin (1:100). Coverslips were mounted in anti-Fade Dapi-Fluoromount-G™ (Southern Biotechnology Associates, Inc., AL). Images in Figs 1A, 4A,B, 5C, and 7E were acquired using a laser-scanning confocal microscope imaging system (LSM510) with a Zeiss Plan-Apochromat 63× /NA1.2 water immersion objective or Plan-Apochromat 100× /NA1.4 oil immersion objective. Images in Figs 3A, 5A,C, 6D, 8A and B were acquired using a Zeiss ApoTome2 microscope imaging system with a Zeiss Plan-Apochromat 100×/NA1.4 oil immersion objective. Images in Figs 6A and 7B were acquired using a Leica epifluorescence microscope with a Leica PL-Fluotar 40×/NA0.7 objective.

### Live Cell Imaging

SrcY527F-transformed NIH3T3 cells transiently expressing GFP-UtrCH or GFP-actin were seeded on 0.17-mm glass coverslips. For Fig. 2B, SrcY527F-transformed NIH3T3 cells transiently expressing mCherry-UtrCH were plated on glass coverslips coated with 20 ng/ml Alexa Fluor 488-conjugated fibronectin for 12 h. Cells were maintained in a microcultivation system with temperature and CO_2_ control devices (Carl Zeiss) and monitored on an inverted microscope (Axio Observer D1, Carl Zeiss) using a Plan-Apochromat 40× objective (NA 0.6 Ph2 korr; Carl Zeiss). Images were captured every 3 min for 24 h using a digital camera (AxioCam MRm D, Carl Zeiss) and analyzed with AxioVision Rel 4.8 software (Carl Zeiss). The wavelength of 450–490 nm was used to excite GFP and Alexa Fluor 488. The 515–565 nm beam path filter was used to acquire images of the emission from GFP and Alexa Fluor 488. The wavelength of 515–560 nm was used to excite mCherry and the 590–650 beam path filter was used to acquire images of the emission from mCherry. The size of the podosome rosette as they started to undergo fission or disassembly was measured with ZEISS ZEN2 image software.

### Statistics

Statistical analyses were performed using Student’s t tests. Differences were considered to be statistically significant at P < 0.05.

## Electronic supplementary material

Supplementary figures
