# Supplementary material for: Biogenesis of podosome rosettes through fission
Source: Sci Rep. 2018 Jan 11;8:524. doi: 10.1038/s41598-017-18861-2 (PMC5765046; doi:10.1038/s41598-017-18861-2)
Supplement: Supplementary file 1 — Supplementary figures [file 41598_2017_18861_MOESM1_ESM.pdf]

Supplementary Information

Biogenesis of podosome rosettes through fission

Szu-Lin Kuo, Chien-Lin Chen, Yi-Ru Pan, Wen-Tai Chiu, and Hong-Chen Chen

Supplementary Figures

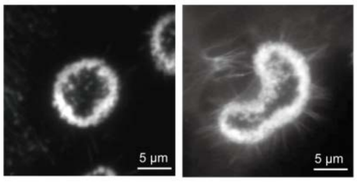

Figure S1. Both circular and concave types of podosome rosettes are in close proximity to the ventral surface of the cell. SrcY527F-transformed NIH 3T3 cells were fixed and stained for actin filaments with phalloidin. Images of podosome rosettes were taken with total internal reflection fluorescence microscopy. Representative images of podosome rosettes with circular or concave shape are shown. The scale bar represents 5 μm.

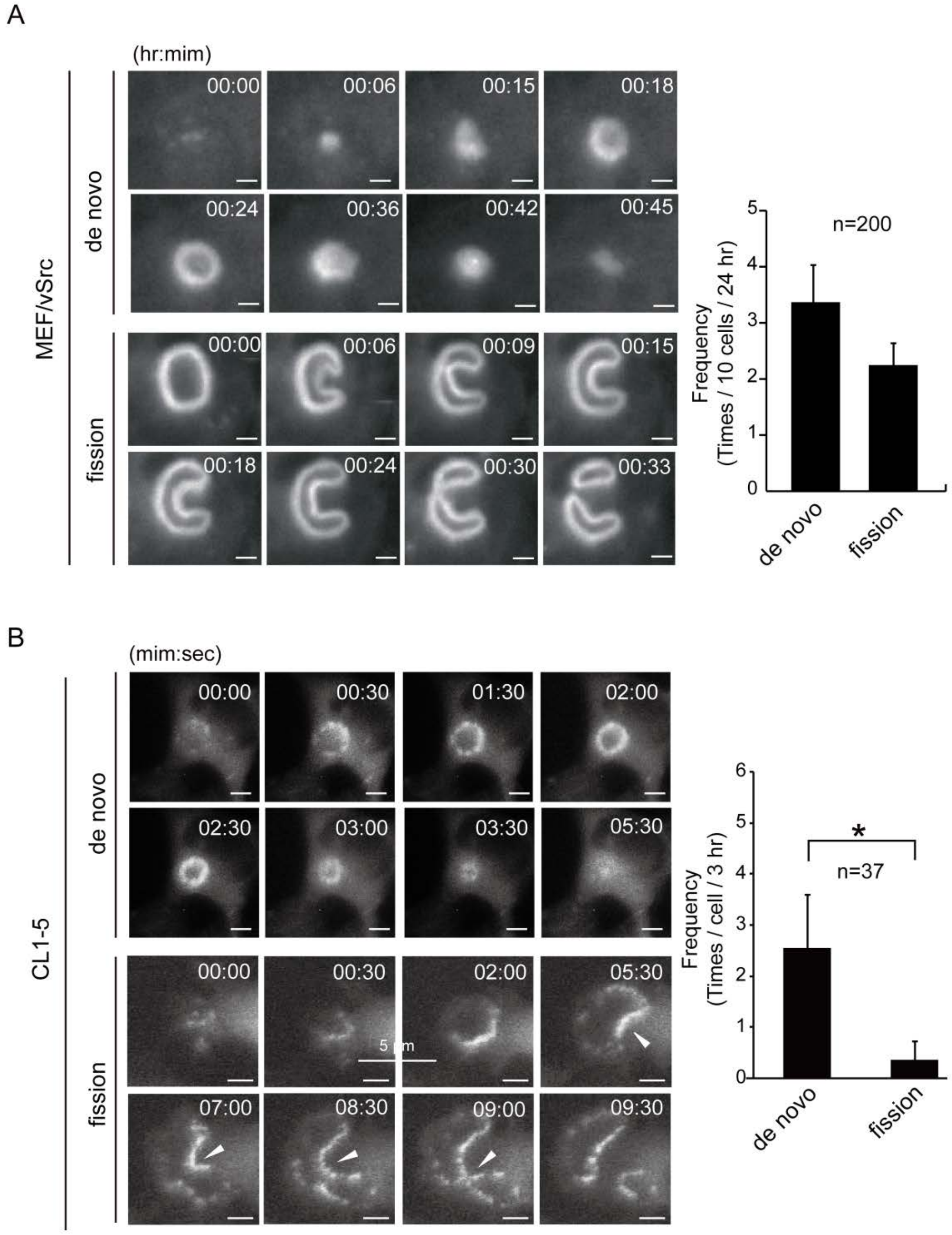

Figure S2. Podosome rosette fission is observed in Src-transformed mouse embryonic fibroblasts and human lung cancer CL1-5 cells. (**A**) GFP-actin was stably expressed in vSrc-transformed mouse embryonic fibroblasts (MEF/vSrc) and the cells were monitored with time-lapse microscopy at 3 min/frame for 24 h. Representative image frames are shown to demonstrate that new podosome rosettes arise through *de novo* assembly or fission in these cells. The scale bar represents 5 μm. The frequencies of the cell to generate podosome rosettes through *de novo* assembly and fission were measured (n=200). Values (means ± s.d.) are from five independent experiments. (**B**) GFP-actin was stably expressed in human lung cancer CL1-5 cells and the cells were monitored with time-lapse microscopy at 30 sec/frame for 3 h. Representative image frames are shown to demonstrate that new podosome rosettes arise through *de novo* assembly or fission in these cells. Arrowhead indicates the cleavage site of the podosome rosette. The scale bar represents 5 μm. The frequencies of the cell to generate podosome rosettes through *de novo* assembly and fission were measured (n=37). Values (means ± s.d.) are from three independent experiments. *P<0.05.

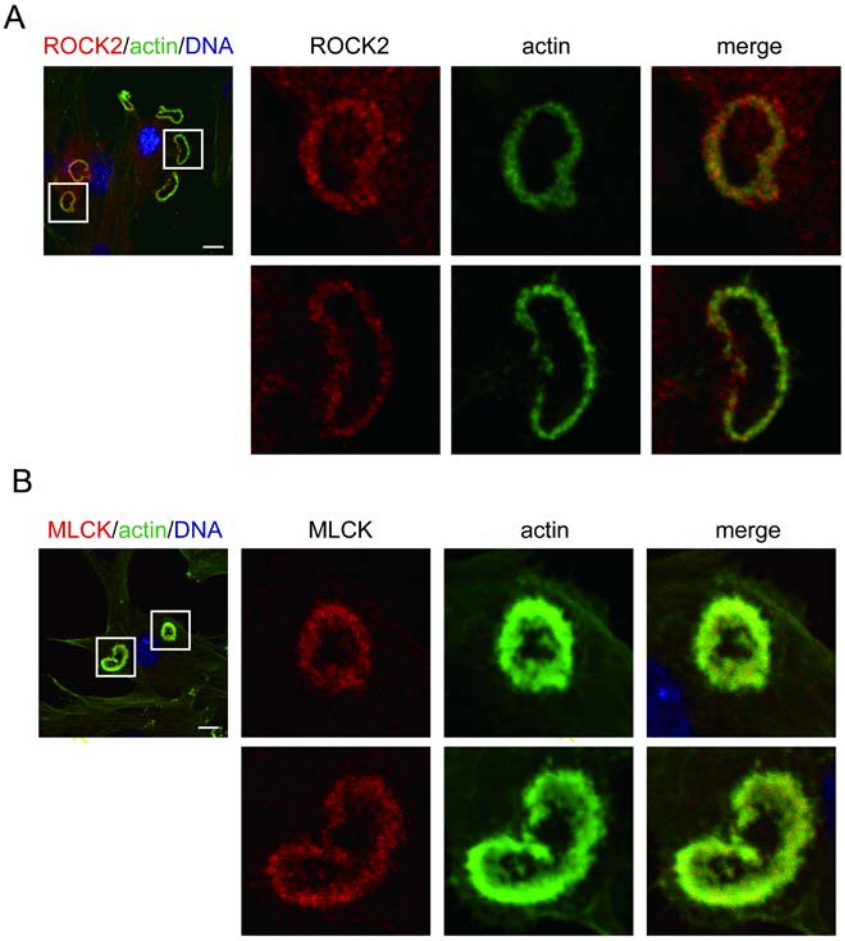

Figure S3. The distributions of ROCK II and MLCK at podosome rosettes. (**a**) SrcY527F-transformed NIH3T3 cells were fixed and stained for actin filaments and ROCK II with phalloidin and anti-ROCK II, respectively. Representative images of podosome rosettes with circular or concave shape are shown. The scale bar represents 10 μm. (**b**) SrcY527F-transformed NIH3T3 cells were fixed and stained for actin filaments and MLCK with phalloidin and anti-MLCK, respectively. Representative images of podosome rosettes with circular or concave shape are shown. The scale bar represents 10 μm.

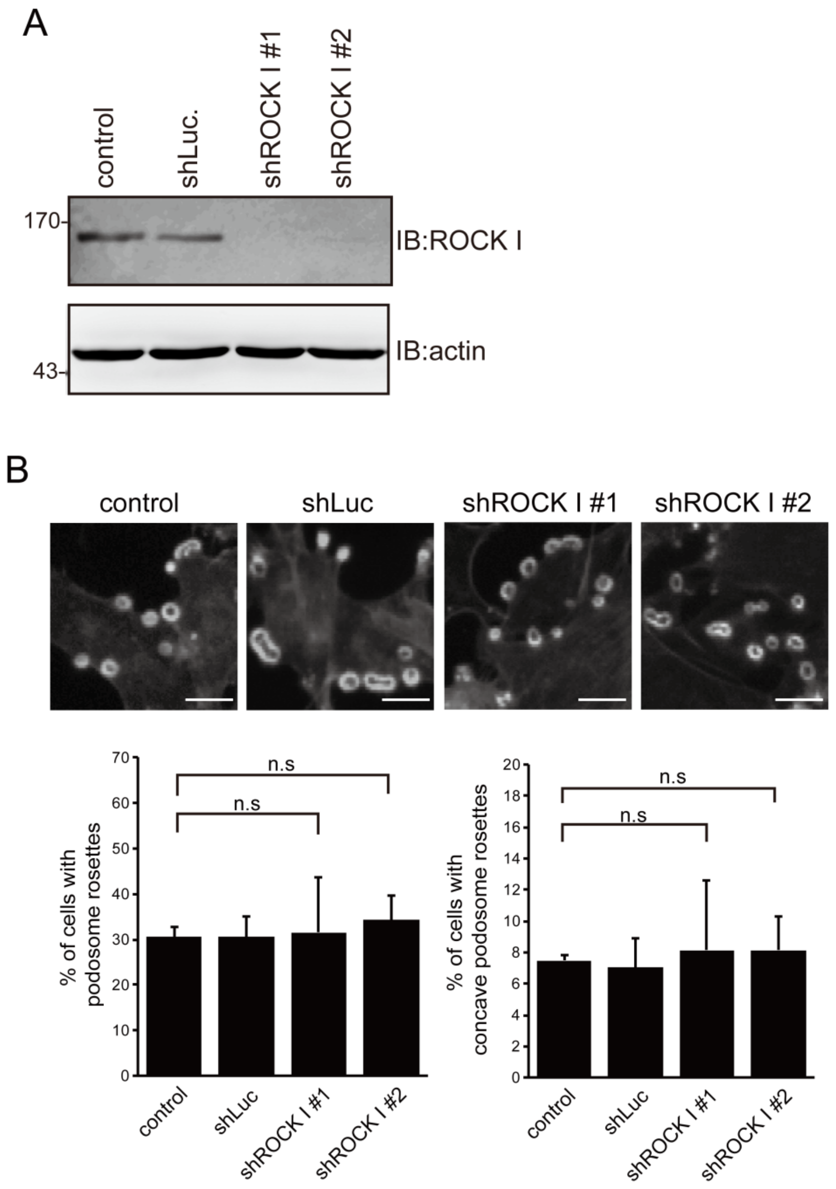

Figure S4. ROCK I is not involved in podosome rosette formation. (**a**) SrcY527F-transformed 3T3 cells were infected with lentiviruses expressing shRNAs specific to ROCK I (#1 and #2), or luciferase as a control and selected in the medium containing puromycin. The cell lysates were analyzed by immunoblotting with anti-ROCK I. (**b**) The cells expressing shRNAs were fixed and stained for actin filaments with phalloidin. Representative images were taken with epifluorescence microscopy. The scale bar represents 20 μm. The percentage of the cells containing podosome rosettes (left) and the percentage of the cells containing concave podosome rosettes (right) were measured (n>1800). The values (means ± s.d.) are from three independent experiments. n.s, no significant differences.
